# Safety and Effectiveness of Intravitreal Brolucizumab Injection in Combination With Sub-Tenon's Capsule Triamcinolone Acetonide Injection for Polypoidal Choroidal Vasculopathy During the Loading Phase

**DOI:** 10.7759/cureus.59481

**Published:** 2024-05-01

**Authors:** Yuji Yoshikawa, Yu Sakaki, Kei Shinoda, Keiko Kataoka

**Affiliations:** 1 Ophthalmology, Saitama Medical University, Saitama, JPN; 2 Ophthalmology, Kozawa Eye Hospital and Diabetes Center, Ibaraki, JPN; 3 Opthalmology, Saitama Medical University, Saitama, JPN; 4 Ophthalmology, Kyorin University, Tokyo, JPN

**Keywords:** age-related macular degeneration, sub-tenon's capsule triamcinolone acetonide injection, polypoidal choroidal vasculopathy, loading phase, intravitreal brolucizumab injection

## Abstract

Background: This study evaluated the safety and effectiveness of combining intravitreal brolucizumab injection with sub-tenon's capsule triamcinolone acetonide injection (STTA) during the loading phase for polypoidal choroidal vasculopathy (PCV).

Methods: In this retrospective observational study, untreated patients with PCV receiving intravitreal brolucizumab injections with STTA during loading at Saitama Medical University Hospital’s Eye Center from May 2021 to June 2022 were analyzed. Complete regression rates of polypoidal lesions were assessed using indocyanine green angiography 12 weeks post-treatment initiation.

Results: Nineteen patients (19 eyes) participated. Best-corrected visual acuity significantly improved at eight weeks compared to baseline. No significant intraocular pressure increases occurred throughout the loading phase, while central foveal and choroidal thickness significantly reduced at 4, 8, and 12 weeks. Subretinal fluid was present in all patients before treatment, rapidly resolving post-intravitreal brolucizumab injections and STTA, with residual rates of 36.8% (seven eyes) and 5.3% (one eye) at four and 12 weeks, respectively. Intraocular inflammation did not occur during the loading phase, and the complete regression rate of polypoidal lesions was 89.5% (17 eyes).

Conclusions: Combining intravitreal brolucizumab injection with STTA during the loading phase may be one treatment option for PCV management.

## Introduction

Polypoidal choroidal vasculopathy (PCV) is a subtype of neovascular age-related macular degeneration (nAMD) [1]. Generally, PCV is more frequent in Asian countries than in Western countries, with a prevalence ranging from 22.3% to 61.6% [2-6]. Currently, the primary treatment for nAMD utilizes anti-vascular endothelial growth factor (VEGF) agents. This category of therapies includes brolucizumab [7-9] and faricimab [10], alongside options such as ranibizumab [11] and aflibercept [12]. Notably, treatment strategies for PCV involve eliminating exudative changes, extending dose intervals, and managing an increased polyp regression rate. Polyp persistence suggests continuous activity [13,14] and can potentially cause severe visual impairment due to rupture-related subretinal hemorrhage [15-17].

Recently, brolucizumab has emerged as a novel therapeutic drug. This drug is a single-chain antibody fragment and the smallest functional unit of an antibody. It is designed to allow the delivery of a more significant molar dose. It potentially shows more effective tissue penetration [7,18,19] and a longer duration of action [20] than larger molecules. The HAWK and HARRIER trials [8] reported extended dosing intervals and high rates of fluid disappearance compared to those with aflibercept [7-9]. Another study has demonstrated a higher polyp regression rate than that with aflibercept [21]. However, using brolucizumab has a higher risk of severe adverse events, notably intraocular inflammation, surpassing the risks associated with other drugs [22]. The heightened risk includes the potential occurrence of occlusive retinal vasculitis occurrence, which can lead to irreversible visual impairment [22]. Although brolucizumab is expected to be an effective therapeutic agent for PCV treatment, intraocular inflammation management, which frequently occurs in the Japanese population [23-31], is a major challenge. In clinical practice, measures are being taken to prevent the onset of intraocular inflammation by the concomitant use of sub-tenon’s capsule triamcinolone acetonide injection (STTA) [23,30].

However, the efficacy and safety of concomitant STTA during the loading phase of PCV treatment have not been studied. The aim of this study was evaluation of the safety and effectiveness of intravitreal brolucizumab injection in combination with STTA for PCV during the loading phase.

## Materials and methods

This retrospective observational study was approved by the Ethics Committee of Saitama Medical University, Iruma, Japan (approval number 2022-044; Date: 1/August/2022). Patients diagnosed with untreated PCV who visited the Eye Center at Saitama Medical University Hospital between May 2021 and June 2022 and received a monthly intravitreal brolucizumab injection with at least one STTA (20 mg) injection during the loading phase were included in the study. The diagnosis of PCV was made by indocyanine green angiography (ICGA) and defined as those with polypoidal lesions by ICGA among those diagnosed with nAMD. Polyps were identified by ICGA as round or oval hyperfluorescent structures. STTA injections were given at the same time as intravitreal brolucizumab injections at the physician's discretion. Intravitreal brolucizumab (6 mg) injections were performed 3.0-4.0 mm posterior to the corneal limbus, and STTA (20 mg) injections were performed through the inferior conjunctival fornix. Data including age, sex, best-corrected visual acuity (BCVA; measured by metric Landholt rings and converted to logarithm of the minimum angle of resolution [logMAR] units), intraocular pressure (IOP) before treatment and at 4, 8, and 12 weeks after treatment initiation, and the presence of intraocular inflammation were retrospectively extracted from medical records.

The presence of intraocular inflammation was determined based on the presence of inflammatory cells in the anterior chamber on the slit-lamp and vitreous opacities on the fundus examination findings as described in the medical record. The retinal vascular changes, such as kyereleis plaques, arterial whitening or occluded arteriole, and presence of vitreous opacity, were assessed with wide-field fundus photographs. Vitreous inflammation was also assessed in the presence of vitreous haze on optical coherence tomography (OCT, Spectralis, Heidelberg Engineering, Germany). We checked the fluorescence angiography examination, which was performed at week 12, and evaluated for retinal vasculitis, including vascular occlusion. All images of each point (0, 4, 8, and 12 weeks) were checked by one retinal specialist.

Using horizontal cross-sectional images passing through the fovea on OCT, we measured the central foveal thickness (CFT) and central choroidal thickness (CCT) before treatment and at 4, 8, and 12 weeks after treatment initiation. Additionally, we investigated the residual fluid rates at each time point from the OCT volume-scan images.

Swept Souse-OCT angiography image (PLEX Elite 9000, version 1.6.0.21130; Carl Zeiss Meditec) including macular neovascularization (MNV) before treatment was captured in Image J software (National Institutes of Health, Bethesda, MD), and the vessel area of MNV was measured. Using ICGA at baseline and 12 weeks after treatment initiation, we evaluated the complete regression rate of the polypoidal lesions.

**Figure 1:**
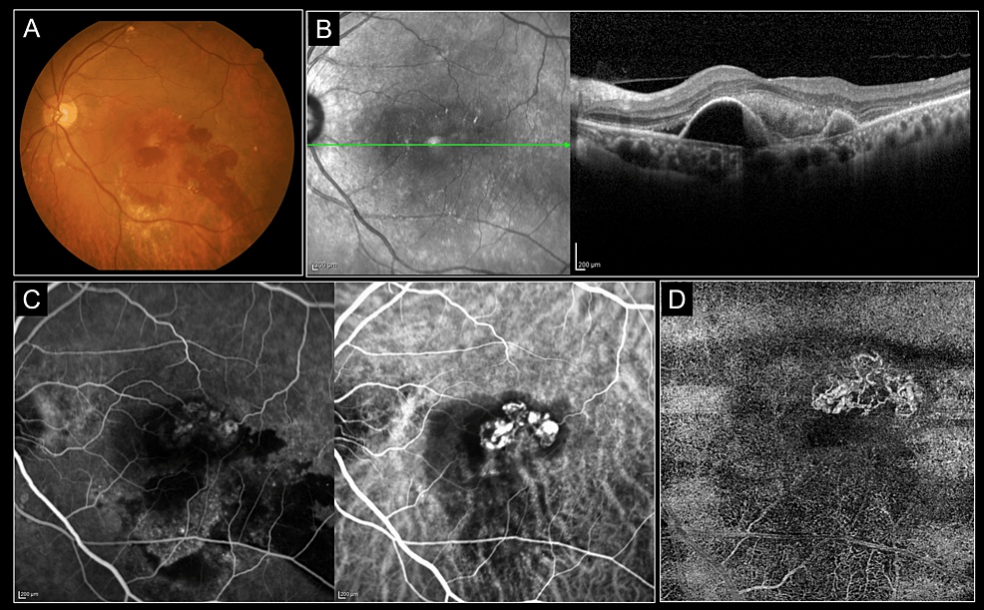
The representative case before treatment (79 years, male) The fundus photograph showed retinal hemorrhage in the macula of the left eye (A). Optical coherence tomography detected subretinal and sub-retinal pigment epithelium (RPE) fluid (B), and optical coherence tomography angiography detected macular neovascularization (C). Fluorescence angiography and indocyanine green angiography detected polypoidal lesions (D).

**Figure 2:**
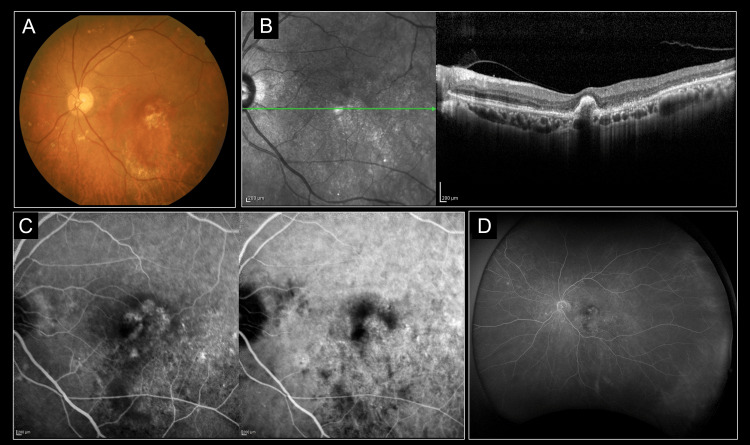
The representative case after loading phase (79 years, male) This case was after the loading phase (at 12 weeks) of the case shown in Figure 1. After the loading phase, retinal hemorrhage decreased in the macula of the left eye and no vitreous opacity was observed (A). Optical coherence tomography showed the disappearance of subretinal and sub-RPE fluid and no vitreous haze. (B) Indocyanine green angiography confirmed complete regression of polypoidal lesions (C). Wide-field fluorescein angiography fundus examination did not detect vasculitis or vascular occlusion (D).

The representative case (79 years, male) of image analysis is shown in Figures 1, 2. The fundus photograph showed retinal hemorrhage in the macula area of the left eye; OCT detected subretinal and sub-retinal pigment epithelium (RPE) fluid, and OCT-angiography detected MNV. ICGA detected polypoidal lesions, which led to the diagnosis of PCV (Figure 1). After the loading phase (at 12 weeks), macular retinal hemorrhage was reduced, and no vitreous opacity was observed; OCT showed the disappearance of subretinal and sub-RPE fluid and no vitreous haze. ICGA showed complete regression of polypoidal lesions, and wide-field fluorescein angiographic fundus examination did not detect vasculitis or vascular occlusion (Figure 2).

Data analysis

Numerical variables are presented as mean ± standard deviation. Pre-treatment (week 0) and post-treatment (weeks 4, 8, and 12) parameters were compared using one-way repeated ANOVA and the paired t-test with Bonferroni adjustment. All statistical analyses were performed using JMP 10.1 (SAS Institute, Cary, NC, USA) and STATA 16 (Stata Corp., College Station, TX, USA) software. Statistical significance was set at P < 0.05 for two-group comparisons and P < 0.017 for three-group comparisons with Bonferroni adjustment.

## Results

A total of 19 patients (19 eyes) were included. The participants included 16 men (84.2%) and three women (15.8%), with an average age of 74.6 ± 7.4 years. Before treatment, the average logMAR BCVA was 0.22 ± 0.29, IOP was 14.1 ± 2.9 mmHg, CFT was 329.2 ± 128.8 μm, and CCT was 243.2 ± 65.1 μm (Table 1). No patient had a history of uveitis or retinal vasculopathy.

**Table 1:** Baseline characteristics (n=19 eyes) Mean ± Standard deviation BCVA, best-corrected visual acuity; IOP, intraocular pressure; CFT, central foveal thickness; CCT, central choroidal thickness; MNV, macular neovascularization.

Characteristics	
Age, years	74.6 ± 7.4
Sex (female/male)	3/16
Ocular parameter	
BCVA (logMAR)	0.21 ± 0.29
IOP (mmHg)	14.1 ± 2.9
CFT (µm)	329.2 ± 128.8
CCT (µm)	243.2 ± 65.1
MNV (mm^2^)	1.14 ± 2.13

**Figure 3:**
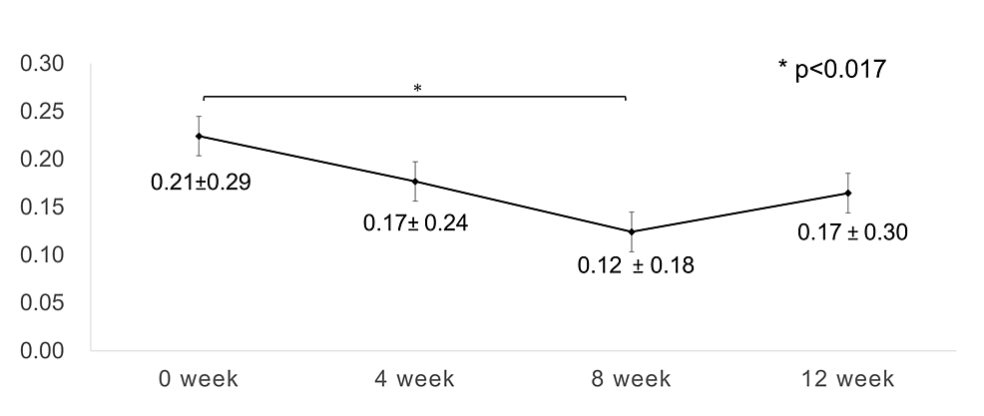
Change in visual acuity during the loading phase Compared with the baseline value, logMAR best-corrected visual acuity showed a significant improvement at the 8-week time point (p=0.0428; one-way repeated ANOVA, p=0.015; paired t-test; a significance was at p < 0.017 for paired t-test with Bonferroni adjustment

On average, patients received STTA injections 1.32 times during the loading phase, ranging from one to three injections. Sixteen eyes (84.2%) received STTA only in the first session, and three eyes (15.7%) received three concomitant injections of STTA during the loading phase at the physician's discretion. Compared with baseline values, BCVA showed a significant improvement at the 8-week time point (p=0.017) (Figure 3).

**Figure 4:**
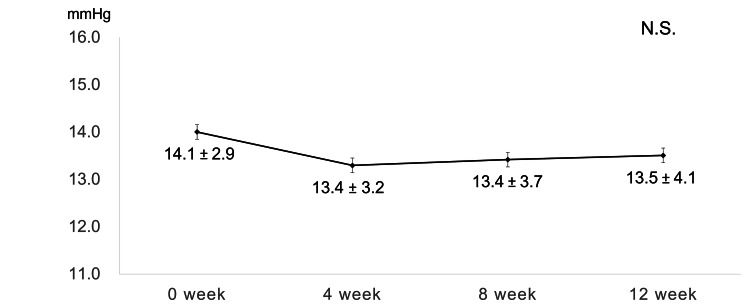
Change in intraocular pressure during the loading phase During the loading phase, no significant increase (mean change; range -4.6 to 7.2) in intraocular pressure, compared to the baseline value, was observed at any time point  (N.S; one-way repeated ANOVA, N.S; paired t-test; a significance was at p < 0.017 for paired t-test with Bonferroni adjustment). N.S., not significant.

There was no subject with cataract progression, and none had cataract surgery during the loading phase. Compared with the baseline value, IOP showed no significant increase (mean change; range -4.6 to 7.2) and did not require additional glaucoma medication and surgical intervention at any time during the loading phase (Figure 4).

**Figure 5:**
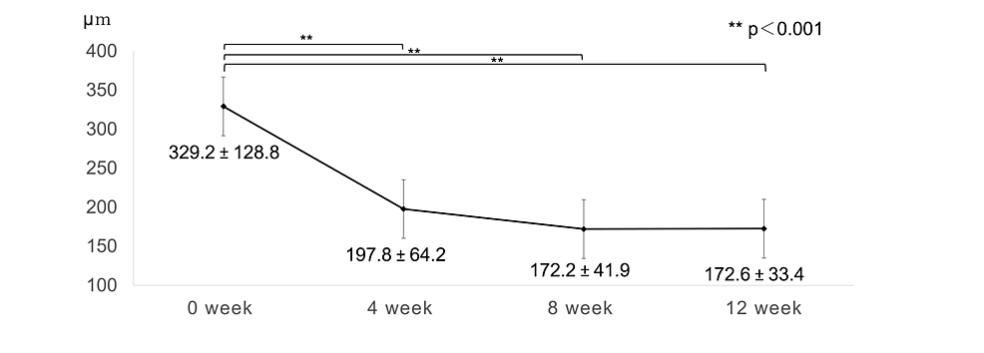
Change in central foveal thickness during the loading phase Central foveal thickness showed a significant decrease at all time points after the start of treatment (p<0.001; one-way repeated ANOVA, p<0.001 respectively; paired t-test; a significance was at p < 0.017 for paired t-test with Bonferroni adjustment); at 12 weeks, it decreased from 329.2 ± 128.8 to 172.6 ± 33.4.

**Figure 6:**
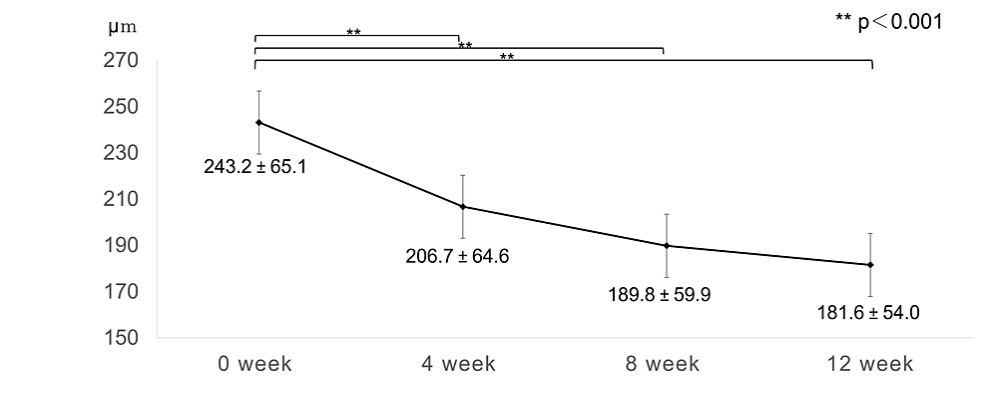
Change in central choroidal thickness during the loading phase Central choroidal thickness showed a significant decrease at all time points after the start of treatment (p<0.001; one-way repeated ANOVA, p<0.001 respectively; paired t-test; a significance was at p < 0.017 for paired t-test with Bonferroni adjustment); at 12 weeks, it decreased from 243.2 ± 65.1 to 181.6 ± 54.0.

Compared with the baseline values, CFT and CCT showed significant reductions at all time points after the start of treatment, with CFT decreasing from 329.2 ± 128.8 to 172.6 ± 33.4 and CCT from 243.2 ± 65.1 to 181.6 ± 54.0 after 12 weeks (Figures 5, 6).

**Figure 7:**
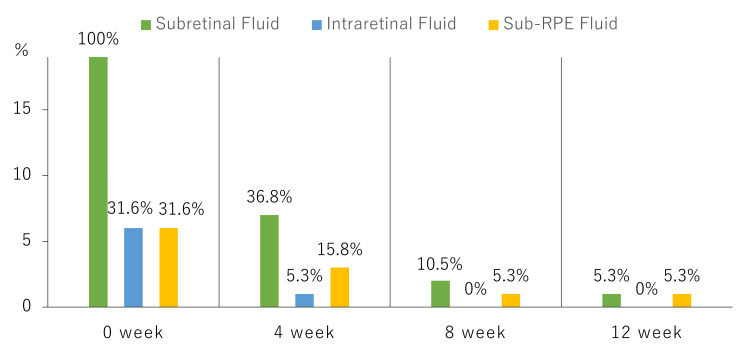
Residual ratio of fluid during the loading phase. Subretinal fluid was present in 100% (19 eyes) of the patients before treatment and resolved quickly after brolucizumab and STTA injections, with a residual rate of 36.8% (seven eyes) and 5.3% (one eye) at four and 12 weeks, respectively. Furthermore, at 12 weeks, the residual ratio of intraretinal fluid was 0%, and that of sub-retinal pigment epithelium fluid was 5%. RPE, retinal pigment epithelium.

Subretinal fluid (SRF) was present in 100% (19 eyes) of the patients before treatment and resolved quickly after intravitreal brolucizumab injection and STTA, with a residual rate of 36.8% (seven eyes) and 5.3% (one eye) at four and 12 weeks, respectively. Intraretinal fluid (IRF) and sub-RPE fluid were each present in 31.6% (six eyes) before treatment. After the start of treatment, IRF disappeared at eight weeks, and the ratio of sub-RPE fluid decreased to 5.3% (one eye) at 12 weeks (Figure 7). Furthermore, the occurrence rate of intraocular inflammation during the loading phase was 0%. ICGA 12 weeks after treatment initiation showed a complete regression rate of 89.5% for polypoidal lesions in the loading phase.

## Discussion

This study demonstrated that the combined intravitreal brolucizumab injection and STTA during the loading phase reduced the incidence of intraocular inflammation and allowed the safe initiation of treatment without causing severe complications. Additionally, the study showed that the complete regression rate of polypoidal lesions treated with intravitreal brolucizumab injection and STTA was comparable to or exceeded the rates reported in previous studies utilizing brolucizumab monotherapy [29,32,33].

One strategy for treating PCV involves improving the regression rate of polypoidal lesions. Existing treatments have reported a regression rate of 23.3% with ranibizumab in the EVEREST II study at three months [11] and 38.9% with aflibercept in the PLANET study at 12 months [12]. Combining ranibizumab with photodynamic therapy has been reported to improve polyp occlusion rates by 71.4% in EVEREST II at three months [11]. However, photodynamic therapy carries risks such as bleeding, choroidal hypoperfusion, and atrophy [34-37]. In previous studies using brolucizumab, Matsumoto et al. (2021) [29], Matsumoto et al. (2022) [31], Fukuda et al. [33], and Ito et al. [32] reported complete regression rates of 72.7-78.6% after loading phase and 93.3% at one year [32], respectively. The present study’s complete regression rate was 89.5% at three months. This suggested that brolucizumab is a promising agent for PCV treatment with a high polyp regression rate, even when used as a combination therapy.

Brolucizumab is also known for its high fluid control capability. A sub-analysis of the HAWK trial targeting PCV showed that brolucizumab more rapidly improved the SRF and/or IRF than did aflibercept; specifically, at the four-week point, SRF persisted at 70% with aflibercept and 49% with brolucizumab [38]. However, our study found an even lower SRF residual rate of 36.8% at the four-week point. Although a direct comparison with previous studies is not possible because the initial SRF levels and the size or properties of polyps were different, it is possible that the anti-inflammatory effect of STTA contributed to the further SRF reduction since nAMD also causes inflammatory changes [39,40].

However, serious side effects have also been reported. The HAWK and HARRIER study reported intraocular inflammation related to brolucizumab in 4.6% of patients overall [22]. In a sub-analysis focusing on Japanese PCV patients within the HAWK and HARRIER studies, the incidence of intraocular inflammation was reported in 15.4% [38]. Furthermore, the incidence of intraocular inflammation in Japanese patients ranges from 9.4% to 28.6% in clinical studies [23-31]. Thus, brolucizumab-related intraocular inflammation is a significant concern for the Japanese population. Notably, recent data from the OCTOPUS and SWIFT studies conducted in Europe revealed the incidence rate of intraocular inflammation of 10.5%[41]. Therefore, the issue of intraocular inflammation is not confined to the Japanese population but is a concern for patients globally. Severe visual impairment can occur if occlusive vasculitis develops owing to intraocular inflammation. Therefore, if intraocular inflammation is detected, more robust anti-inflammatory treatments such as sub-tenon's capsule injection or systemic medication are required in addition to topical steroid eye drops [41,42]. However, it is challenging to predict and/or prevent intraocular inflammation before the injection of brolucizumab. Therefore, prevention of intraocular inflammation is essential. In a previous case report, a patient who developed intraocular inflammation after brolucizumab monotherapy was successfully treated with an intravitreal brolucizumab injection and a small STTA dose (5 mg) without recurrence of intraocular inflammation [23]. In addition, Hikichi reported that combining STTA with brolucizumab reduced the incidence of intraocular inflammation by 28.6% (four of 14 eyes) to 0% (0 of 30 eyes) when switching from another anti-VEGF agent for nAMD [30]. This study, which was limited to untreated PCV cases, similarly demonstrated that safe initiation of brolucizumab treatment could be achieved by combining STTA during the loading phase. Previous studies have reported a history of retinal vasculitis and/or retinal occlusion and female sex as risk factors for brolucizumab-related intraocular inflammation [43]. The fact that our study did not include any patients with a history of retinal vasculitis and/or retinal occlusion among the subjects and the low proportion of females (15.8%) may contribute to the low incidence of intraocular inflammation [43]. However, despite males being more commonly affected by nAMD in Japan [44,45], the incidence of intraocular inflammation is elevated among Japanese patients compared to other racial groups. Hence, the revelation that none of the 19 cases, albeit with a small representation of females, exhibited intraocular inflammation is a noteworthy outcome. In addition, due to the low incidence of vascular occlusion without intraocular inflammation, it remains challenging to determine if STTA effectively averted vascular occlusion without intraocular inflammation within the confines of such a limited study cohort.

Since most patients received only one session of STTA during the loading phase, STTA during the loading phase may be sufficient using only the initial dose. Although a previous study reported that 81.1% of intraocular inflammation-related adverse events occurred during the loading phase [41], this study did not provide insights into the frequency of STTA administration during the maintenance phase. A study evaluating the efficacy of triamcinolone for uveitic macular edema reported that 36 of 73 eyes (49%) required additional doses for eight weeks after the initial dose for six months [46]. Thus, managing inflammation during the maintenance phase may be necessary after the loading phase. Further longitudinal studies are required for elucidation.

Concerns related to STTA use include potential increases in IOP, long-term risks of cataracts, and infections [35,47]. Previous studies using brolucizumab in combination with STTA have reported a 10% incidence of transient IOP increase following STTA administration, which resolved without the need for additional drug therapy [30]. While our study focused on short-term observation during the loading phase and did not encounter severe side effects or unmanageable IOP elevations, it should be noted that this was a retrospective study with a limited number of participants, and there was no control group receiving brolucizumab monotherapy. All images at each point (0, 4, 8, and 12 weeks) were checked by a single retina specialist, but because this was a retrospective study, it is possible that minor intraocular inflammation that could not be detected in the clinical examinations or by each image was not detected. Therefore, large-scale prospective comparative studies are needed to validate the preventive effect of STTA in the future.

## Conclusions

In this study, there are no intraocular inflammation among study populations with intravitreal brolucizumab injection in combination with STTA during the loading phase. Combining intravitreal brolucizumab injection with STTA during the loading phase may be one treatment option for PCV management. However, nAMD treatment spans an extended period, and managing intraocular inflammation during maintenance remains challenging. Repeated STTA administration may increase the occurrence of side effects; therefore, the number and administration methods should be determined cautiously. Due to the small sample size of this study, large-scale, long-term studies are necessary in the future.
